# Supplementary material for: Energy saving strategy of cloud data computing based on convolutional neural network and policy gradient algorithm
Source: PLoS One. 2022 Dec 30;17(12):e0279649. doi: 10.1371/journal.pone.0279649 (PMC9803140; doi:10.1371/journal.pone.0279649)
Supplement: Attachment — Submitted filename: Response to reviewers.docx [file pone.0279649.s003.docx]

| **Date:** | Oct 06 2022 11:18AM |
| --- | --- |
| **To:** | "Dexian Yang" yangdexian@stu.xju.edu.cn |
| **From:** | "PLOS ONE" plosone@plos.org |
| **Subject:** | PLOS ONE Decision: Revision required [PONE-D-22-22823] |

PONE-D-22-22823

Energy Saving Strategy of Cloud Data Computing Based on Convolutional Neural Network and Policy Gradient Algorithm

PLOS ONE

Dear Dr. Yang,

Thank you for submitting your manuscript to PLOS ONE. After careful consideration, we feel that it has merit but does not fully meet PLOS ONE’s publication criteria as it currently stands. Therefore, we invite you to submit a revised version of the manuscript that addresses the points raised during the review process.

Please submit your revised manuscript by Nov 20 2022 11:59PM. If you will need more time than this to complete your revisions, please reply to this message or contact the journal office at [plosone@plos.org](mailto:plosone@plos.org). When you're ready to submit your revision, log on to https://www.editorialmanager.com/pone/ and select the 'Submissions Needing Revision' folder to locate your manuscript file.

Please include the following items when submitting your revised manuscript:

- A rebuttal letter that responds to each point raised by the academic editor and reviewer(s). You should upload this letter as a separate file labeled 'Response to Reviewers'.
- A marked-up copy of your manuscript that highlights changes made to the original version. You should upload this as a separate file labeled 'Revised Manuscript with Track Changes'.
- An unmarked version of your revised paper without tracked changes. You should upload this as a separate file labeled 'Manuscript'.

If you would like to make changes to your financial disclosure, please include your updated statement in your cover letter. Guidelines for resubmitting your figure files are available below the reviewer comments at the end of this letter.

If applicable, we recommend that you deposit your laboratory protocols in protocols.io to enhance the reproducibility of your results. Protocols.io assigns your protocol its own identifier (DOI) so that it can be cited independently in the future. For instructions see: [https://journals.plos.org/plosone/s/submission-guidelines#loc-laboratory-protocols](https://journals.plos.org/plosone/s/submission-guidelines#loc-laboratory-protocols). Additionally, PLOS ONE offers an option for publishing peer-reviewed Lab Protocol articles, which describe protocols hosted on protocols.io. Read more information on sharing protocols at <https://plos.org/protocols?utm_medium=editorial-email&utm_source=authorletters&utm_campaign=protocols>.

We look forward to receiving your revised manuscript.

Kind regards,

Osamah Shihab Albahrey
Academic Editor
PLOS ONE

Journal Requirements:

When submitting your revision, we need you to address these additional requirements.

1. Please ensure that your manuscript meets PLOS ONE's style requirements, including those for file naming. The PLOS ONE style templates can be found at 
https://journals.plos.org/plosone/s/file?id=wjVg/PLOSOne_formatting_sample_main_body.pdf and 
https://journals.plos.org/plosone/s/file?id=ba62/PLOSOne_formatting_sample_title_authors_affiliations.pdf

2. Please note that PLOS ONE has specific guidelines on code sharing for submissions in which author-generated code underpins the findings in the manuscript. In these cases, all author-generated code must be made available without restrictions upon publication of the work. Please review our guidelines at https://journals.plos.org/plosone/s/materials-and-software-sharing#loc-sharing-code and ensure that your code is shared in a way that follows best practice and facilitates reproducibility and reuse.

3. Thank you for stating in your Funding Statement: 
"This research was supported by the National Natural Science Foundation of China under Grant Nos. 61862060."

Please provide an amended statement that declares *all* the funding or sources of support (whether external or internal to your organization) received during this study, as detailed online in our guide for authors at http://journals.plos.org/plosone/s/submit-now.  Please also include the statement “There was no additional external funding received for this study.” in your updated Funding Statement. 
Please include your amended Funding Statement within your cover letter. We will change the online submission form on your behalf.

4. Thank you for stating the following financial disclosure: 
"This research was supported by the National Natural Science Foundation of China under Grant Nos. 61862060."

Please state what role the funders took in the study. If the funders had no role, please state: ""The funders had no role in study design, data collection and analysis, decision to publish, or preparation of the manuscript."" 
If this statement is not correct you must amend it as needed. 
Please include this amended Role of Funder statement in your cover letter; we will change the online submission form on your behalf.

5. PLOS requires an ORCID iD for the corresponding author in Editorial Manager on papers submitted after December 6th, 2016. Please ensure that you have an ORCID iD and that it is validated in Editorial Manager. To do this, go to ‘Update my Information’ (in the upper left-hand corner of the main menu), and click on the Fetch/Validate link next to the ORCID field. This will take you to the ORCID site and allow you to create a new iD or authenticate a pre-existing iD in Editorial Manager. Please see the following video for instructions on linking an ORCID iD to your Editorial Manager account: https://www.youtube.com/watch?v=_xcclfuvtxQ

6. Please include captions for your Supporting Information files at the end of your manuscript, and update any in-text citations to match accordingly. Please see our Supporting Information guidelines for more information: http://journals.plos.org/plosone/s/supporting-information.

Additional Editor Comments:

The reviewers have commented on the paper. This paper could be published if you revised it carefully considering all given comments.


[Note: HTML markup is below. Please do not edit.]

Reviewers' comments:

Reviewer's Responses to Questions

**Comments to the Author**

1. Is the manuscript technically sound, and do the data support the conclusions?

The manuscript must describe a technically sound piece of scientific research with data that supports the conclusions. Experiments must have been conducted rigorously, with appropriate controls, replication, and sample sizes. The conclusions must be drawn appropriately based on the data presented.

Reviewer #1: Yes

Reviewer #2: Yes

2. Has the statistical analysis been performed appropriately and rigorously?

Reviewer #1: Yes

Reviewer #2: N/A

3. Have the authors made all data underlying the findings in their manuscript fully available?

The [PLOS Data policy](http://www.plosone.org/static/policies.action#sharing) requires authors to make all data underlying the findings described in their manuscript fully available without restriction, with rare exception (please refer to the Data Availability Statement in the manuscript PDF file). The data should be provided as part of the manuscript or its supporting information, or deposited to a public repository. For example, in addition to summary statistics, the data points behind means, medians and variance measures should be available. If there are restrictions on publicly sharing data—e.g. participant privacy or use of data from a third party—those must be specified.

Reviewer #1: No

Reviewer #2: Yes

4. Is the manuscript presented in an intelligible fashion and written in standard English?

PLOS ONE does not copyedit accepted manuscripts, so the language in submitted articles must be clear, correct, and unambiguous. Any typographical or grammatical errors should be corrected at revision, so please note any specific errors here.

Reviewer #1: Yes

Reviewer #2: Yes

5. Review Comments to the Author

Please use the space provided to explain your answers to the questions above. You may also include additional comments for the author, including concerns about dual publication, research ethics, or publication ethics. (Please upload your review as an attachment if it exceeds 20,000 characters)

Reviewer #1: This work combined Deep Reinforcement Learning and neural networks to reduce the energy consumption of task scheduling in Cloud Data Computing data centers. The work sounds good and can be published after minor revision.
Comments:
-Lack of references in the introduction section. Many issues mentioned without reference.

Reply: Thank you for your comment. According to the introduction, supplementary references have been provided. See References 1, 3, 4, 7, 8, 9, 11 for details.
-For reader convenience, I suggest that the notations should be listed with one table with their meanings

Reply: Thank you for your comment. Table 1 has been cited to illustrate the characteristics of relevant scholars' research.
-The data in the excel sheet need to be specified more clearly.

Reply: Thank you for your comment. For a detailed description of the data in excel, see the raw file.
-Please add a link (URL) to the dataset which you used.

Reply: Thank you for your comment. This article uses links to datasets explained in Section 3.5.

Reviewer #2: My Comments for the work are as follows,

==== INTRODUCTION ====

The authors need to better explain the context of this research, including why the research problem is important.
Reply: Thank you for your comment. The research background has been explained in detail in the second paragraph of the introduction section. In addition, for the research purpose of this article, it has been explained in the third paragraph of the introduction section.

The introduction should clearly explain the key limitations of prior work that are relevant to this paper.
Reply: Thank you for your comment. In the introduction section, the differences between the research of this paper and previous research work, as well as the shortcomings of previous research on energy planning and energy management have been explained, leading to the research of this paper. See the fourth paragraph of the introduction section.

Contributions should be highlighted more. It should be made clear what is novel and how it addresses the limitations of prior work.
Reply: Thank you for your comment. The research contribution of this paper has been clarified in the introduction section, and the innovation of this paper has been explained, as well as the advantages of cloud data computing task scheduling model based on policy gradient algorithm in this paper, which solves the energy consumption problem of cloud data computing. Please refer to the fourth paragraph of the introduction section for details.

==== RELATED WORK ====

The authors should explain clearly what the differences are between the prior work and the solution presented in this paper.
Reply: Thank you for your comment. The differences between this study and previous studies have been explained in detail. See the second paragraph of Section 2.2 for details.

The authors should add a table that compares the key characteristics of prior work to highlight their differences and limitations. The authors may also consider adding a line in the table to describe the proposed solution.
Reply: Thank you for your comment. A table has been added in Section 2.2 to explain the characteristics of previous research methods and the advantages of this research method. See Table 1 for details.

==== METHOD ====

The authors should first give an overview of their solution before explaining the details.
Reply: Thank you for your comment. The research process of this paper has been explained in the research method section. Please refer to the first paragraph of Section 3.1 for details.

It is important to clearly explain what is new and what is not in the proposed solution. If some parts are identical, they should be appropriately cited and differences should be highlighted.
Reply: Thank you for your comment. References have been added to the first paragraph of Section 3.1 to highlight the innovation of this research method. Please refer to the first paragraph of Section 3.1 for details and references 29, 30.

The authors should add proof(s) of the properties, theorem or lemmas contained in the paper.
Reply: Thank you for your comment. In this paper, the process of convolutional neural network algorithm has been explained in Equations 1 to 10, and the derivation process of strategy gradient algorithm has been supplemented in Section 3.2. Please refer to current Equations 12 to 16 for details.

==== EXPERIMENTS ====

There is not enough discussion of the experimental results.

Reply: Thank you for your comment. The research results of this paper have been discussed based on the research of scholars. Please refer to "5. Discussion" for details.

6. PLOS authors have the option to publish the peer review history of their article ([what does this mean?](https://journals.plos.org/plosone/s/editorial-and-peer-review-process#loc-peer-review-history)). If published, this will include your full peer review and any attached files.


If you choose “no”, your identity will remain anonymous but your review may still be made public.


**Do you want your identity to be public for this peer review?** For information about this choice, including consent withdrawal, please see our [Privacy Policy](https://www.plos.org/privacy-policy).

Reviewer #1: No

Reviewer #2: No

[NOTE: If reviewer comments were submitted as an attachment file, they will be attached to this email and accessible via the submission site. Please log into your account, locate the manuscript record, and check for the action link "View Attachments". If this link does not appear, there are no attachment files.]

While revising your submission, please upload your figure files to the Preflight Analysis and Conversion Engine (PACE) digital diagnostic tool, <https://pacev2.apexcovantage.com/>. PACE helps ensure that figures meet PLOS requirements. To use PACE, you must first register as a user. Registration is free. Then, login and navigate to the UPLOAD tab, where you will find detailed instructions on how to use the tool. If you encounter any issues or have any questions when using PACE, please email PLOS at [figures@plos.org](mailto:figures@plos.org). Please note that Supporting Information files do not need this step.

*In compliance with data protection regulations, you may request that we remove your personal registration details at any time. [(Remove my information/details)](https://www.editorialmanager.com/pone/login.asp?a=r). Please contact the publication office if you have any questions.*
