# Supplementary material for: Energy saving strategy of cloud data computing based on convolutional neural network and policy gradient algorithm
Source: PLoS One. 2022 Dec 30;17(12):e0279649. doi: 10.1371/journal.pone.0279649 (PMC9803140; doi:10.1371/journal.pone.0279649)
Supplement: Attachment — Submitted filename: second revision.docx [file pone.0279649.s004.docx]

**Second Review report of paper entitled:**

**Energy Saving Strategy of Cloud Data Computing Based on Convolutional Neural Network and Policy Gradient Algorithm**

This work combined Deep Reinforcement Learning and neural networks to reduce the energy consumption of task scheduling in Cloud Data Computing data centers. The work sounds good and can be published after minor revision.

Second revision

Lack of references in the introduction section. Many issues mentioned without reference. (Solved )

- For reader convenience, I suggest that the notations should be listed with one table with their meanings

Author answer: Thank you for your comment. Table 1 has been cited to illustrate the characteristics of relevant scholars' research.

- **By list of notations I mean something like this:**

- Please add a link (URL) to the dataset which you used.

Author answer: Thank you for your comment. This article uses links to datasets explained in Section 3.5.

**There is no links to data in section 3.5.**
